# Atomistic Simulation and Micro-Pillar Compression Studies on the Influence of Glass–Glass Interfaces on Plastic Deformation in Co-P Metallic Nano-Glasses

**DOI:** 10.3390/ma18081853

**Published:** 2025-04-17

**Authors:** Yongwei Wang, Jiashu Chen, Mo Li, Guangping Zheng

**Affiliations:** 1 Collaborative Innovation Center of Steel Technology, University of Science and Technology Beijing, Beijing 100083, China; yw_wang@ustb.edu.cn; 2 Department of Mechanical Engineering, Hong Kong Polytechnic University, Hung Hom, Kowloon, Hong Kong, China; jiashu.chen@polyu.edu.hk; 3 School of Materials Science and Engineering, Georgia Institute of Technology, Atlanta, GA 30332, USA

**Keywords:** metallic nanoglass, shear bands, glass–glass interfaces, molecular dynamics, AC-TEM

## Abstract

The glass–glass interfaces (GGIs) play an important role during the plastic deformation of metallic nano-glasses (NGs) such as Sc-Fe NGs. In this work, Co-P nano-glasses are synthesized by pulse electrodeposition. Their mechanical properties are characterized by micro-pillar compression and compared to those obtained by molecular dynamics (MD) simulation. The MD simulation reveals that the GGIs with a particular incline angle (about 50.0°) in the direction of applied uniaxial strain is preferable for the accommodation of localized plastic deformation in NGs. The results are consistent with those obtained by spherical aberration-corrected transmission electron microscopy, which reveals that most of shear bands form an angle of about 58.7° to the direction of compressive strain applied on the Co-P micro-pillar. The phenomena are explained with the differences in chemical composition and atom diffusion in the glassy grain interiors and in the GGI regions. This work sheds some light on the deformation mechanisms of NGs and provides guidelines for designing NGs with improved mechanical properties.

## 1. Introduction

Metallic glasses (MGs), which are characterized by a lack of long-range order and possess unique properties such as high specific strength, good corrosion resistance, soft magnetism, and unique net-shape formability, have attracted tremendous research interests since their discovery in the 1960s [1]. However, the plastic deformation of MGs at temperatures well below the glass transition temperature is highly localized within a narrow region, the so-called shear band, resulting in catastrophic failure and limiting the structural reliability of MGs and their practical applications [2,3,4,5,6]. In recent years, enormous efforts have been devoted to improving the plasticity of MGs. The most intuitive route is to prevent the propagation of primary shear bands through MGs. The concept of heterogeneous structure is widely accepted as useful for improving the plasticity of MGs by blocking the localized shear bands [7,8,9,10,11,12,13,14]. For instance, the introduction of a second phase or inclusions, surface treatments, or mechanical deformations such as shot peening or grinding in the surface region can induce the formation of heterogeneous structures [15,16,17,18]. The introduction of heterogeneity into MGs can avoid the formation of penetrating shear bands and improve the deformability of MGs [19,20,21,22,23,24].

The manipulation of the structural heterogeneity in conventional metallic glasses, as produced by rapid quenching, is challenging. Nano-glass (NG), a new type of amorphous material with microstructures, was first proposed by Jing and Gleiter et al. in 1989 [25,26]. The microstructure of NG consists of nano-sized glassy grains (<100 nm), connected by loose glass–glass interfaces (GGIs). The GGI is usually about several nanometers wide with a locally reduced density relative to the densities in the interior of the glassy grains. These heterogeneous microstructures enable NG to possess excellent mechanical properties. Recent studies have demonstrated that the mechanical properties of NGs can be tailored by controlling the size of the glassy grains and GGIs [27,28]. Notably, the glass–glass interfaces containing excess free volumes can result in soft regions, which serve as potential sites for shear bands. This heterogeneous structure plays a crucial role in regulating shear-banding kinetics and preventing strains from localizing within dominant shear bands. The GGIs facilitate the nucleation of multiple shear bands, thereby avoiding shear localization and improving tensile ductility [29]. The increase in ductility is intimately connected to the underlying heterogeneous microstructure. It is suggested that the interfaces, as weak regions, can be critical microstructures that tune the mechanical properties of NGs. The high density of free volumes in the GGI regions also contributes to the strength softening. Although the mechanical properties and deformation mechanisms were investigated through quantitative ex situ compression tests on micron-sized samples [23,30,31], and as there have been studies on the mechanical behavior of NGs, a profound understanding of the role of GGIs on plastic flow behavior has not obtained.

Currently, NGs are mainly produced by inert gas condensation, magnetic sputtering, and severe plastic deformation. Compared to these complicated and high-cost synthesis routes, electrodeposition is a low-cost and facile technique with which synthesizes high-purity NGs. Many preparation parameters, especially peak current density, frequency, and duty cycle, could be adjusted in electrodeposition [32,33]. In this study, we conduct the electrodeposition of Co-P NG thick films, controlling their chemical composition and microstructure by changing the electrodeposition parameters such as duty cycles and voltages. The microstructure and heterogeneity of chemical compositions of the free-standing films are characterized. The influence of GGIs on the plastic deformation in NGs is investigated by compressive tests on the micro-pillar of Co-P samples, which are further compared to those as obtained by molecular dynamics (MD) simulation.

## 2. Experimental and Computational Methods

### 2.1. Experimental Methods

Samples of Co-P NGs were fabricated by pulse electrodeposition in a three-electrode electrochemical cell at room temperature. The NG films were deposited on a titanium substrate, which was used as a working electrode. A graphite rod and a saturated calomel electrode were used as counter electrode and reference electrode, respectively. The electrolyte was an aqueous solution of CoSO_4_·7H_2_O (0.1 mol/L), C_6_H_5_Na_3_O_7_·2H_2_O (0.2 mol/L), H_3_BO_3_ (0.5 mol/L), and NaH_2_PO_2_·H_2_O (0.2 mol/L), which was adjusted to have a pH value of 2–3 by adding concentrated H_2_SO_4_. A working potential of 3.8 V, at each duty cycle of a pulse voltage (with a duty cycle of 30% at 100 kHz), was applied to the working electrode for 2 h. A Teflon mold was used to prepare films in a rectangular shape (30 mm × 3.5 mm × 0.05 mm), which were mechanically exfoliated from the titanium substrate for use in further experimental studies.

The NG films mechanically exfoliated from the titanium substrate were characterized by X-ray diffraction (XRD, Rigaku Smartlab, Rigaku, Tokyo, Japan). Scanning electron microscopy (SEM, TESCAN VEGA3, TESCAN, Brno, Czech Republic) was used to observe the nanostructure of the samples. Chemical composition was evaluated by atom probe tomography (APT, LEAP 5000HR, CAMECA, Gennevilliers, France). High-resolution images of glassy grains with sizes less than 30 nm in diameter were taken by transmission electron microscopy (TEM, JOEL JEM-2011, JOEL, Tokyo, Japan). Spherical aberration-corrected transmission electron microscopy (AC-TEM, FEI Titan3, Hillsboro, OR, USA) was used to observe the atomic structure of the deformed Co-P NGs. A micro-pillar of Co-P NGs was prepared using a focus ion beam (FIB, Thermo Scientific Helios 5 UX DualBeam, Thermo Fisher Scientific, Waltham, MA, USA). Compression was carried out on the micro-pillar using a nano-indentation instrument (Agilent, Santa Clara, CA, USA, Nano indenter G200).

### 2.2. Computational Methods

The large-scale atomic/molecular massively simulator (LAMMPS) software (version 3 Mar 2020) is used for molecular dynamics (MD) simulations [34]. For the Co-P alloy studied in this work, we employ the Pak–Doyama-type potentials to describe the inter-atomic Co-P, Co-Co, and P-P interactions [35]. The dependence of the potential energy (*E_ij_*) between *i* and *j* atoms, measured in eV, on the distance (*r*), measured in Å, is described as follows:

(1) $$E_{\mathrm{Co}\text{-}\mathrm{Co}}\left(r\right)=-0.12812{\left(r-1.82709\right)}^{4}+1.15421{\left(r-2.50849\right)}^{2}-0.13448,\;r<3.34\;Å$$

(2) $$E_{\mathrm{Co}\text{-}P}\left(r\right)=-0.15374{\left(r-1.58709\right)}^{4}+1.38505{\left(r-2.26849\right)}^{2}-0.13167,\;r<3.20\;Å$$

(3) $$E_{P\text{-}P}\left(r\right)=-0.07435{\left(r-2.60709\right)}^{4}+0.64791{\left(r-3.27885\right)}^{2}-0.07531,\;r<3.34\;Å$$

where *r* is the distance between atom *i* and atom *j*. When *r* exceeds the cutoff distance, *E_ij_* is set to be 0 eV. Previous studies derived the amorphous atomic structure of Co-P glass via MD simulation using Equations (1)–(3) for *E_ij_*. The calculated structure aligns well with experimental observations [36,37], affirming the accuracy and reliability of these inter-atomic potentials for simulating the mechanical properties of Co-P NGs. Periodic boundary conditions are applied along the *x-*, *y-*, and *z*-directions, with the numerical integration step for the equations of motion set to 1 fs during the simulations. A Nose–Hoover thermostat is used to regulate the temperature (300~550 K) of the simulated supercell.

**Figure 1:**
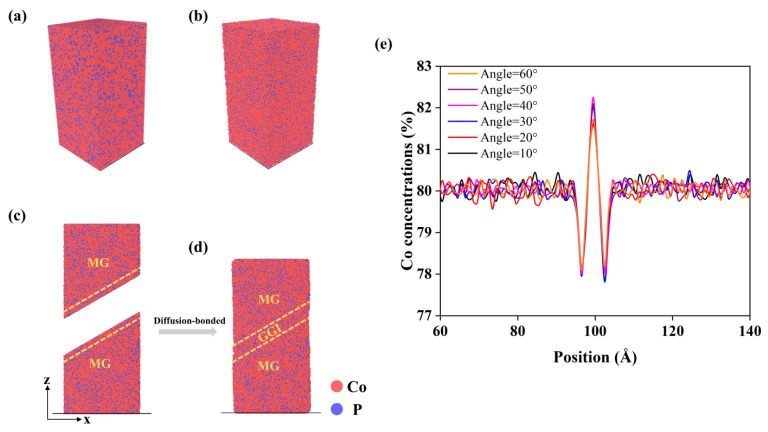
(**a**) The initial Co_80_P_20_ model system with an FCC structure. (**b**) The Co_80_P_20_ MG system generated via MD simulation. (**c**,**d**) Schematics of the formation of an NG system in MD simulation, which consists of two MG blocks and a glass–glass interface with an incline angle to the *xy*-plane of *a_x_* = 30°; atoms in the GGI region are depicted by dashed lines. (**e**) The distribution of element concentrations along the direction perpendicular to the GGI in the Co_80_P_20_ MG system shown in (**d**). Red balls represent Co atoms, while blue balls represent P atoms.

Initially, as shown in Figure 1a, we established a rectangular supercell with dimensions of 11 × 11 × 21.6 nm, where Co and P atoms, in a ratio of 80:20, are placed in a face-centered cubic (FCC) lattice. Subsequently, the Co_80_P_20_ alloy was relaxed at 300 K. Then, we increased the system temperature to 2000 K until the alloy was melted completely and became liquid. The entire system was then rapidly cooled to 300 K at a rate of 10^10^ K/s, resulting in the formation of Co_80_P_20_ metallic glass (MG) with distinct glass phases. Although the cooling rate in MD is much higher than that used in the melt-quenching preparation of MGs, the as-obtained glass phases are consistent with those of NG prepared by electrodeposition. The MG was cut into a cylindrical shape with a diameter of 10 nm and an aspect ratio of 2:1 along the *z*-direction, and the size effects on the simulation results are mainly related to the aspect ratio of NG systems. Subsequently, in the middle of the MG cylinder, the MG was further cut along a plane with an incline angle of *a_x_* (*a_x_* = 10°, 20°, 30°, 40°, 50°, 60°) to the cross sectional *xy*-plane (or 90-*a_x_* to the *z*-direction), forming two identical MG blocks. The blocks were separated and then fully relaxed in NVT MD simulations at a temperature of 300 K, creating two free surfaces in the MG blocks, and the MG blocks were heated to 400 K for 20 ns until their free surfaces reached equilibrium, as shown in Figure 1c. Next, the MG blocks were put together at their surfaces, which became a GGI through the diffusion bonding of the MG blocks. The MD simulation of the diffusion bonding processes was carried out using an NPT ensemble with a hydrostatic pressure of 1 bar and at an annealing temperature of 550 K (below glass transition temperature, *T*_g_~560 K). Finally, the entire system was cooled to 300 K, resulting in an NG system that was composed of two MG blocks, separated by a glass–glass interface with an incline angle *a_x_*, as shown in Figure 1d.

## 3. Results and Discussion

### 3.1. The Effects of a GGI with an Inclining Angle a_x_ on the Mechanical Properties of NGs

The effects of a GGI with an incline angle *a_x_* on the mechanical properties of NGs are investigated by MD simulation. After the GGI is formed in the NG system, it typically has a thickness of about 2 nm, as marked by the dashed lines in Figure 1d. The atomic concentrations in the GGI region along the direction perpendicular to the interface are determined to analyze the composition heterogeneity. Figure 1e shows that the atomic concentration of Co in the GGI region is influenced by the angle *a_x_*. For instance, when *a_x_* increases from 10° to 40°, the Co content (*X*_Co_) at the center of the GGI region increases from 81.56% to 82.15%. *X*_Co_ decreases to 81.71% when *a_x_* increases from 40° to 60°. Therefore, the highest *X*_Co_ occurs in the system with a GGI incline angle of 40°, which exhibits the largest gradient of Co content or the strongest composition heterogeneity across the GGI, as shown in Figure 1e.

The self-diffusion coefficient can be calculated from the long-time limit of the mean squared displacement (MSD) using the Einstein relation [38]:

(4) $$D=\frac{1}{6}\underset{t\to \infty }{lim}\left[\frac{d}{\mathrm{dt}}\left\langle \left[\overset{\to }{r}{\left(t\right)}^{2}\right]\right\rangle \right]$$

Based on Equation (4), the diffusion coefficients of Co and P atoms are determined by fitting MSD versus time *t* to a linear function in the 0–20 ps time frames.

**Figure 2:**
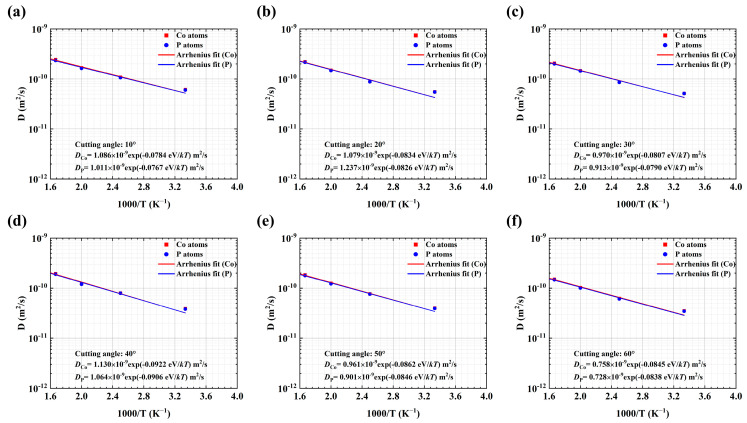
The diffusion coefficients *D* of Co (red square) and P (blue dot) atoms at GGIs with different incline angles: (**a**) 10°, (**b**) 20°, (**c**) 30°, (**d**) 40°, (**e**) 50°, (**f**) 60°. The blue and red straight lines represent curve fitting, using the Arrhenius formula for P and Co atoms, respectively.

**Figure 3:**
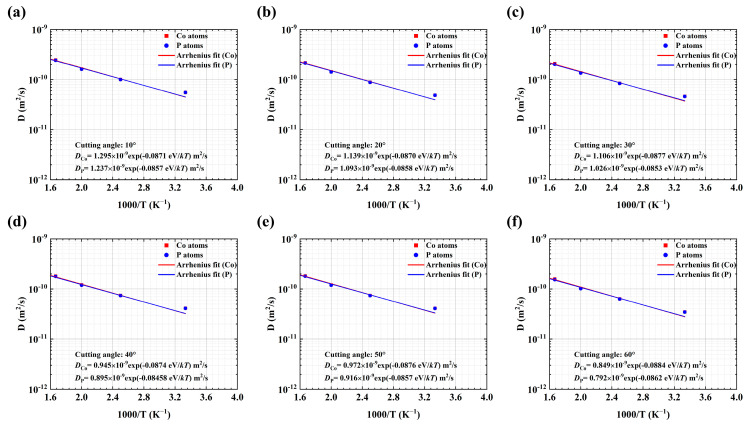
The values of the diffusion coefficient *D* of Co (red square) and P (blue dot) atoms in the interior of MG blocks in the NG system with different GGI incline angles of (**a**) 10°, (**b**) 20°, (**c**) 30°, (**d**) 40°, (**e**) 50°, and (**f**) 60°. The blue and red straight lines represent curve fitting using the Arrhenius formula for P and Co atoms, respectively.

The diffusion coefficients of Co and P atoms in the NG system at various temperatures are plotted in Figure 2 and Figure 3. As anticipated, the diffusion coefficients increase with increasing temperature at 300~550 K. It is well-established that the temperature-dependent diffusion coefficient adheres to an Arrhenius-type relationship.

(5) $$D=D_{0}\exp\left(-\frac{E_{a}}{k_{B}T}\right)$$

where *D*_0_ is the diffusion pre-exponential factor and *E_a_* is the activation energy. The parameters *D*_0_, *D,* and *E_a_* for the diffusion of Co and P atoms in the GGI region and interiors of MG blocks are separately determined by Equations (4) and (5), as shown in Table 1.

Generally, the diffusivity of Co is greater than that of P, as indicated in Figure 2 and Figure 3 and listed in Table 1, suggesting that there could be Co enrichment (*X*_Co_ > 80%) at the center of GGIs after the diffusion bonding processes because of the fast diffusion of Co into the GGI region, as shown in Figure 1e. Table 1 indicates that in the NG systems with a GGI incline angle of *a_x_,* the diffusion coefficient of Co or P atoms at 300 K in the GGI region decreases with increasing *a_x_*, except for the NG system with *a_x_* = 40°. Meanwhile, as shown in Table 1, the activation energy of Co or P atoms in the GGI region is lower than that of those atoms in the interiors of MG blocks of an NG system, except for the NG system with *a_x_* = 40°. The results suggest that the *a_x_*-dependent diffusion of atoms in the GGI region could be abnormal at *a_x_* = 40°. In particular, the activation energy of Co or P atoms in the NG system with *a_x_* = 40° is the largest among those in the NG system with different values of *a_x_*. As a result, abnormal composition (as shown in Figure 1e) and atomic structures may exist in the GGI regions with an incline angle of *a_x_* = 40° after the NG systems are formed through the diffusion bonding processes.

**Figure 4:**
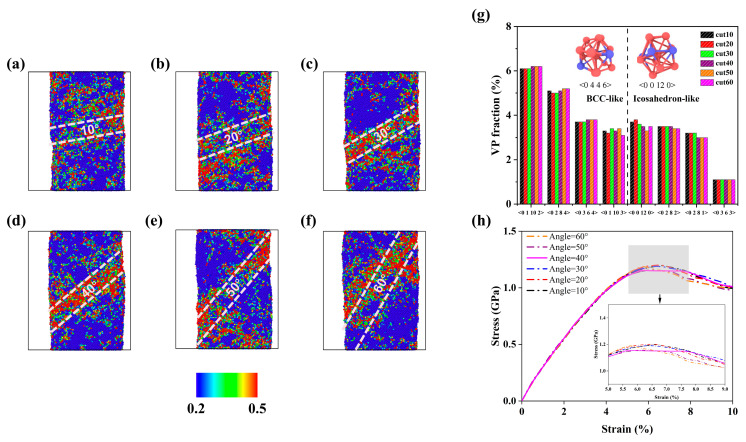
The color plots for atomic shear strains of the Co_80_P_20_ NG systems with GGI incline angles *a_x_* of (**a**) 10°, (**b**) 20°, (**c**) 30°, (**d**) 40°, (**e**) 50° and (**f**) 60° under an applied compressive strain of 10%. The local shear strains of 0.2 or greater are plotted, with the color bar shown at the bottom. The GGI region is indicated by two dashed lines. (**g**) The VP fractions of the GGI region in the NG systems with various *a_x_*. The insets show the atomic structure of typical VPs, indicating that the Co contents for <0 0 12 0> and <0 4 4 6> VPs are 9/12 and 10/13, respectively. (**h**) The compressive stress vs. strain curves for the systems with various angles *a_x_*.

Figure 4a–f depicts the shear bands within the NG systems with different GGI incline angles. The shear transformation zone (STZ), also referred to as the embryonic shear bands, is defined as a region where the atomic shear strains under the plastic deformation are 0.2 or greater [39,40,41]. Embryonic shear bands primarily form and expand in the interiors of MG blocks. However, a shift in the localization of STZ is observed. As the GGI incline angle (*a_x_*) increases from 10° to 40°, the STZ is more likely to occur in the GGI region, indicating that the GGI with increased Co enrichment (increased *X*_Co_) is favorable for the formation of shear bands. In the NG system with *a_x_* = 40°, the embryonic shear band clearly presents in the GGI region, as shown in Figure 4d. This feature of shear banding could be closely related to the strong fluctuation in chemical composition near the center of the GGI region, where there exists a large gradient of Co content, as shown in Figure 1e.

Table 1 lists the fraction of Voronoi polyhedrons (VPs) in the GGI region, which are described using the Voronoi index <n3 n4 n5 n6>, with n3, n4, n5, and n6 representing the number of faces with respective edges of 3, 4, 5, and 6 in the VPs. Notably, the icosahedron-like VPs and the body-centered cubic (bcc)-like VPs have coordination numbers (n3 + n4 + n5 + n6) of 11–12 and 13–14, respectively, which are shown in Figure 4g. It is worth noting that typical icosahedral VPs <0 0 12 0> have relatively lower Co content (0.75) than bcc-like VPs (>0.77), as shown in the insets in Figure 4g. As listed in Table 1, it is evident that in the NG system with *a_x_* = 40°, the fraction of bcc-like VPs in the GGI region is relatively high, while the fraction of icosahedral VPs is relatively low. Therefore, in the NG system with *a_x_* = 40°, the lower proportion of icosahedral VPs in the GGI region may be related to the large Co content *X*_Co_ at the GGI.

Previous studies have shown that the presence of fewer icosahedral VPs in MGs results in lower shear resistance and mechanical strength, making them more prone to plastic deformation under external stresses [39,42,43,44]. Consequently, the mechanical strength of the NG system decreases with increasing composition heterogeneity near the GGI, i.e., higher Co content *X*_Co_ at the center of the GGI region, primarily due to the reduced proportion of icosahedral VPs. Hence, composition heterogeneity significantly affects the mechanical properties of NG systems. Figure 4h shows the stress–strain curves of the NG systems with different *a_x_* subjected to uniaxial compression along the *z*-direction. At a strain rate of 3 × 10^7^ s^−1^, the strain at the maximum stress for the NG systems increases with increasing compositional heterogeneity, as evidenced by the highest *X*_Co_ at the center of GGI region in the NG system with *a_x_* = 40°, although the mechanical strength of the NG system, as measured by the maximum stress, is relatively low.

### 3.2. Micro-Pillar Compression on Co-P Nano-Glasses

**Figure 5:**
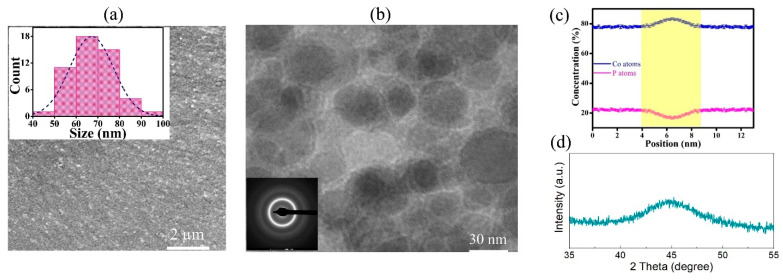
(**a**) SEM images of the Co-P NG sample; the inset shows the histogram plots of sizes of glassy grains. (**b**) TEM images of the sample; the inset shows the SAED patterns. (**c**) The distribution of elemental compositions along the direction perpendicular to GGI, and (**d**) XRD patterns for the Co-P sample.

The SEM and TEM images of Co-P NG samples, as prepared by pulse electrodeposition, are shown in Figure 5a,b, respectively. The average size of glassy grains (D_avg_) is estimated using the histogram plots of grain sizes, as shown in the inset in Figure 5a, which is about 68 nm. The distribution of elemental compositions along the direction perpendicular to a GGI in the Co-P NG sample is shown in Figure 5c, indicating that there is Co enrichment in the GGI regions with a thickness of about 2 nm. The inset in Figure 5b shows the selected area electron diffraction (SAED) patterns, indicating the amorphous nature of Co-P, which is consistent with the XRD patterns of the sample (Figure 5d).

**Figure 6:**
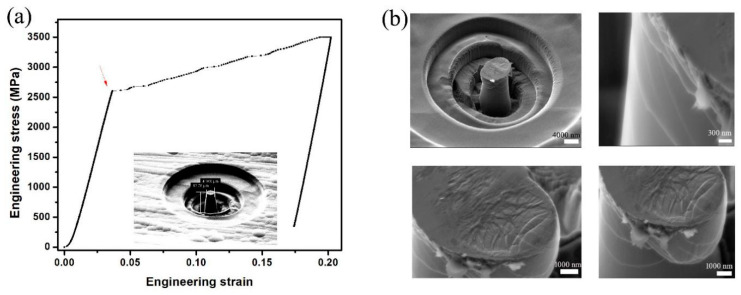
(**a**) The stress–strain relation for the compression test on the micro-pillar sample of Co-P NG (inset). The arrow indicates the yield strength. (**b**) SEM images of the surfaces of fractured samples with cracks.

The Co-P micro-pillar samples are prepared by FIB milling. As shown in Figure 6a, the micro-pillar with a taper angle of 6.1° has a height of 12.78 μm and its upper surface has a diameter of 4.968 μm. A flat indenter, with a 20 μm diameter top surface, is used to compress the micro-pillar sample by applying forces linearly increased with time. Figure 6b shows the stress–strain curve for the compression test performed on the micro-pillar sample, where the taper angle correction of the stresses was performed using the empirical relation proposed in Ref. [45]. Remarkably, typical pop-ins in the stress–strain curve can be observed at the stage of strain hardening when the applied stress exceeds the yield strength of about 2.6 GPa, which last until the sample fractures at a strain of 18.9%. It is worth noting that the stress–strain curves for compression tests on the micro-pillar samples of bulk metal glasses (BMG) generally exhibit serration at applied stresses larger than the yield strength, manifesting little plasticity in BMGs [2,3]. The strain hardening behaviors, as indicated by the stress–strain curve for the micro-pillar sample of Co-P NG, suggest that the shear band propagation seems to be restricted in Co-P NG under plastic deformation because of the existence of nanostructures in the NG (Figure 5b).

**Figure 7:**
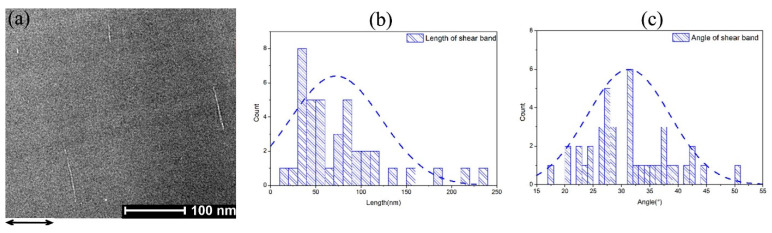
(**a**) AC-TEM images of fractured a micro-pillar sample of Co-P NG with distinct segments of shear bands; the arrow indicates the direction of applied compressive strain. (**b**,**c**) Histogram plots of length and orientation angle of shear bands observed in the AC-TEM images. The dashed lines are fitting curves.

The shear bands in the fractured sample are analyzed by SEM and AC-TEM. Figure 6b shows that buckling occurs in the sample under an applied stress higher than the yield strength, while no definite fracture surface can be determined since the micro-cracks proliferate and extend to the buckled regions. Typical AC-TEM images of a slab with a thickness of 50 nm, cut from the fractured sample, are shown in Figure 7a.

As shown in Figure 7a, shear bands can be distinguished well from the disordered structure of NG. The length of the shear band and its incline angle θ to the cross section of the micro-pillar (perpendicular to the direction of applied compressive strain indicated by the arrow in Figure 7a) can be measured. These were determined for more than 40 shear bands observed in the sample using AC-TEM. Figure 7b,c shows the histogram plots for the length and incline angle of shear bands observed, respectively. It is found that the mean values of shear band length and incline angle are D_M_ = 72.2 nm and θ_M_ = 31.3°, respectively.

Compared to the MD simulation results on the shear banding in Co-P NG with different GGI incline angles *a_x_*, it is found that there is consistency between D_avg_ (68 nm) and D_M_, and between *a_x_* (about 30°–40° as shown in Figure 4c,d for the occurrence of shear bands mainly within the GGI regions) and θ_M_. In the nanostructure of Co-P NG samples prepared by pulse electrodeposition, the adjacent glassy grains could possess GGIs with random orientations, as indicated in Figure 5b. During the plastic deformation in the NG samples, shear banding tends to occur in the GGI regions with a GGI incline angle close to 40°, which could be driven by an applied compressive stress lower than that used for the occurrence of shear banding in the interiors of glassy grains, as demonstrated by the MD simulation results (Figure 4a–f,h). Therefore, shear bands with an incline angle θ of ~40° to the cross section of micro-pillars (or ~50° to the direction of applied compressive strain) could be predominant in the NG sample under plastic deformation, whose characteristic length could be restricted by the sizes of glassy grains as measured by D_avg_.

It is worth noting that the preference of shear banding in the GGI regions with a particular GGI incline angle originates from the composition heterogeneity in the GGI regions in NGs, as elucidated by the MD simulation. The confirmation of such preference of shear banding by the micro-pillar compression experiments suggests that shear banding in NGs under plastic deformation could be controlled to occur in the GGI regions by modulating the chemical compositions of GGIs, which could be facilely implemented by applying heat treatment to NGs at different annealing temperatures or heating/cooling rates. Therefore, this work provides guidelines for designing NGs with improved mechanical properties.

## 4. Conclusions

Co-P NGs are synthesized by pulse electrodeposition at room temperature. They possess nanostructures containing glassy grains with an average size of 68 nm and GGIs with random orientations. The influences of GGI orientation on the plastic deformation of NGs are investigated by molecular dynamics simulation and compressive tests on the NGs with a pillar shape. The results suggest that GGIs with a particular incline angle of about 50.0°–58.7° in the direction of applied compressive strain are preferable for the accommodation of the localized plastic deformation in the NGs. These phenomena are explained by the differences in chemical composition and atom diffusion in the glassy grain interiors and in the GGI regions, as revealed by MD simulation. This work sheds some light on the deformation mechanisms of NGs and provides guidelines for designing NGs with improved mechanical properties. For example, NG wires with a bamboo-like structure where the GGIs have an incline angle of about 50.0°–58.7° to the length direction could possess excellent ductility.

## Figures and Tables

**Table 1:** The diffusion pre-exponential factor *D*_0_, activation energy *E*_a_, and diffusion coefficient *D* (at T = 300 K) of Co and P atoms in the interiors of MG blocks (MG) and GGI region (GGI), and fraction of Voronoi polyhedrons (VPs) in the GGI region, as obtained from MD simulation of the NG systems with different GGI incline angles.

GGI Incline Angle (°)	Region	*D*_0_ (10^−9^ m^2^/s)		*E*_a_ (eV)		*D* (10^−11^ m^2^/s) at T = 300 K		BCC-like VPs (%)	Icosahedron <0,0,12,0> (%)
		Co	P	Co	P	Co	P		
10	GGI	1.0861	1.0112	0.0784	0.0767	6.1490	6.0229	18.2	3.7
	MG	1.2952	1.2371	0.0871	0.0857	5.5274	5.5119		
20	GGI	1.0793	1.2374	0.0834	0.0829	5.5524	5.4942	18.0	3.8
	MG	1.1391	1.0928	0.0870	0.0858	4.8682	4.8477		
30	GGI	0.9701	0.9126	0.0807	0.0790	5.1572	5.1374	18.2	3.6
	MG	1.1057	1.0255	0.0877	0.0853	4.6282	4.5856		
40	GGI	1.1301	1.0642	0.0922	0.0906	3.9001	3.8102	18.4	3.5
	MG	0.9453	0.8948	0.0874	0.0858	4.1331	4.0989		
50	GGI	0.9606	0.9012	0.0862	0.0846	3.9960	3.9357	18.6	3.3
	MG	0.9720	0.9161	0.0876	0.0857	4.1142	4.0908		
60	GGI	0.7579	0.7276	0.0845	0.0838	3.5312	3.4485	18.3	3.5
	MG	0.8489	0.7922	0.0884	0.0862	3.4833	3.4529		

## Data Availability

No new data were created or analyzed in this study.
